# Peroxiredoxin 1 alleviates oxygen-glucose deprivation/ reoxygenation injury in N2a cells via suppressing the JNK/caspase-3 pathway

**DOI:** 10.22038/IJBMS.2023.71390.15528

**Published:** 2023

**Authors:** Yang Yuan, Hongchen Tan, Huailong Chen, Jiawen Zhang, Fei Shi, Mingshan Wang, Gaofeng Zhang, Haipeng Wang, Rui Dong

**Affiliations:** 1 Department of Anesthesiology, Qingdao Municipal Hospital, Qingdao, Shandong, China; 2 Malvern College Qingdao, Qingdao, Shandong, China; 3 Department of Anesthesiology, Qingdao Eight People’s Hospital, Qingdao, Shandong, China; 4 Department of Anesthesiology, Qingdao Clinical College Affiliated to Nanjing Medical University, Qingdao, Shandong, China; 5 Department of Anesthesiology, Weifang No.2 People’s Hospital, Weifang, Shandong, China

**Keywords:** Caspase-3, Cell hypoxia, c-Jun N-terminal kinase, Mice, Neuroblastoma, Peroxiredoxin 1, Reperfusion injury

## Abstract

**Objective(s):**

Cerebral ischemia/reperfusion (I/R) injury inevitably aggravates the initial cerebral tissue damage following a stroke. Peroxiredoxin 1 (Prdx1) is a representative protein of the endogenous antioxidant enzyme family that regulates several reactive oxygen species (ROS)-dependent signaling pathways, whereas the JNK/caspase-3 proapoptotic pathway has a prominent role during cerebral I/R injury. This study aimed to examine the potential mechanism of Prdx1 in Neuro 2A (N2a) cells following oxygen–glucose deprivation and reoxygenation (OGD/R) injury.

**Materials and Methods:**

N2a cells were exposed to OGD/R to simulate cerebral I/R injury. Prdx1 siRNA transfection and the JNK inhibitor (SP600125) were used to interfere with their relative expressions. CCK-8 assay, flow cytometry, and lactate dehydrogenase (LDH) assay were employed to determine the viability and apoptosis of N2a cells. The intracellular ROS content was assessed using ROS Assay Kit. Real-time quantitative reverse transcription polymerase chain reaction (qRT-PCR) and western blot analyses were conducted to detect the expression levels of Prdx1, JNK, phosphorylated JNK (p-JNK), and cleaved caspase-3.

**Results:**

Firstly, Prdx1, p-JNK, and cleaved caspase-3 expression were significantly induced in OGD/R-exposed N2a cells. Secondly, the knockdown of Prdx1 inhibited cell viability and increased apoptosis rate, expression of p-JNK, and cleaved caspase-3 expression. Thirdly, SP600125 inhibited the JNK/caspase-3 signaling pathway and mitigated cell injury following OGD/R. Finally, SP600125 partially reversed Prdx1 down-regulation-mediated cleaved caspase-3 activation and OGD/R damage in N2a cells.

**Conclusion:**

Prdx1 alleviates the injury to N2a cells induced by OGD/R via suppressing JNK/caspase-3 pathway, showing promise as a potential therapeutic for cerebral I/R injury.

## Introduction

Stroke is a common life-threatening cerebrovascular disease in adults resulting in long-term disability and imposing an economic burden on society (1), of which ischemic stroke accounts for approximately 70% of the case (2). Revascularization therapies for cerebrovascular embolization usually cause subsequent destruction of the ischemic brain tissue, which is identified as cerebral ischemia/reperfusion (I/R) injury (3). Oxidative stress plays a crucial role in the complex pathophysiological mechanisms of I/R injury during the revascularization phase (4). Excess accumulation of reactive oxygen species (ROS) is primarily responsible for oxidative stress, which could induce the expression of multiple endogenous protective factors to maintain the survival of nerve cells (5). However, while ROS overwhelms the anti-oxidant defense, mitochondrial autophagy, intracellular apoptosis, and necrosis are unavoidable (6). Therefore, ameliorating oxidative stress would contribute to cerebral protection following reperfusion. 

Peroxiredoxin 1 (Prdx1), as a representative protein of the endogenous anti-oxidant enzyme family, functions as a scavenger of the detrimental ROS and balances the reduction–oxidation status of the cells (7). In response to the remarkable increase in oxidative stress in the focal cerebral I/R model of rats or oxygen–glucose deprivation and reoxygenation (OGD/R) model of rat cortical neurons, Prdx1 expression was correspondingly elevated (8). Additionally, the c-Jun N-terminal kinase (JNK) signaling pathway participated in multiple stress reactions (9). Enhancing the anti-oxidant capacity of brain tissue would inhibit the activation of the JNK/caspase-3 signaling pathway, thereby alleviating brain damage (10, 11). Moreover, preliminary research in the OGD/R model of H9C2 cells showed that Prdx1 inhibited apoptosis by down-regulating the p-JNK/JNK ratio (12).

However, the detailed molecular mechanisms of Prdx1 in cerebral I/R injury remain unknown. We established the OGD/R model of N2a cells to imitate cerebral I/R injury. The Prdx1 siRNA and JNK inhibitor were used to examine the expression of Prdx1, JNK/p-JNK, and cleaved caspase-3, and several cellular injury indicators were tested. The purpose of this study is to explore the protective effects of Prdx1 via the JNK/caspase-3 pathway in OGD/R-exposed N2a cells.

## Materials and Methods

***N2a cells culture and OGD/R model establishment***

The mouse N2a cells were provided by the National Collection of Authenticated Cell Cultures (Shanghai, China). As described previously (13), the cells were kept in a modified incubator containing 5% CO_2_ at 37 ℃ and cultured in Dulbecco’s modified Eagle’s medium (DMEM) supplemented with 10% fetal bovine serum (Hyclone, Beijing, China) and 100 μg/ml penicillin/streptomycin. To mimic the pathological change of cerebral I/R, the OGD/R model of N2a cells was developed as previously described (14). Briefly, N2a cells were incubated in DMEM with normal glucose, and then placed in a sealed hypoxic chamber with a premixed gas (5% CO_2_ and 95% N_2_) for 8 hr at 37 ℃. Subsequently, the cells were perfused by glucose-free fresh DMEM medium under normal normoxic conditions for 24 hr.

***Cell transfection and/or SP600125 treatment***

The siRNA target-specific binding with Prdx1 and its negative control siRNA (siNC) were synthesized by RiboBio (Guangdong, Guangzhou, China). The siRNA sequences that target Prdx1 are shown in Table 1. N2a cells in a 6-well plate were transiently transfected with synthesized siRNA using riboFECT^TM^ CP Transfection Kit (Guangdong, Guangzhou, China) according to the manufacturer’s instructions for 48 hr in siPrdx1 + OGD/R group. After the validation of the transfection efficiency was successful, the OGD/R model was developed. According to the previous study (11), N2a cells were treated with 10 µmol/l of SP600125 (JNK inhibitor) for 24 hr at the initiation of reoxygenation in the OGD/R + SP600125 group and siPrdx1 + OGD/R + SP600125 group.

***Quantitative real-time reverse transcription polymerase chain reaction (qRT-PCR)***

Prdx1 mRNA expression was quantified by qRT-PCR, and the primer sequences (Sangon Biotech, Shanghai, China) used for the amplification reaction are shown in Table 1. The Trizol reagent (Sigma–Aldrich, St Louis, MO, USA) was employed to extract total mRNA from N2a cells, and the isolated mRNA concentration was determined by ScanDrop Ultra-micro spectrophotometer (Jena Analytical Instruments AG, Jena, Germany), thereafter, reverse transcription of mRNA was performed to synthesize template cDNA using PrimeScript^TM^ RT reagent kit with gDNA Eraser (Takara Bio Inc. Beijing, China). The synthesis of cDNA molecules was unwound and amplified by qRT-PCR reaction using a TB Green Premix Ex Taq II kit (Takara Bio Inc. Beijing, China) in T100™ Thermal Cycler Real-Time PCR System (Bio-Rad, Hercules, CA, USA) with the following thermal cycling conditions: 95 °C, 30 sec; 40 cycles at 95 °C, 5 sec; 60 ℃, 30 sec; and 72 ℃, 1 min. GAPDH was set as an internal control for normalizing Prdx1 expression. The 2^−ΔΔCt^ of the data was calculated according to the Ct value measured by qRT-PCR.

***Lactate dehydrogenase (LDH) assay***

The LDH level in the supernatants of N2a cells was measured using an LDH Cytotoxicity Assay kit (Thermofisher Scientific) in accordance with the standard protocols. Briefly, the wells of N2a cells were divided into the following four types: blank control wells (medium without N2a cells), spontaneous LDH release wells (N2a cells untreated with OGD/R), maximum LDH release wells (N2a cells untreated with OGD/R was used for subsequent lysis), and experimental LDH release wells (N2a cells exposed to OGD/R). After centrifugation, the supernatant culture medium of all the wells in the 96-well plate was collected. Then 60 μl supernatant was transferred into a new 96-well plate, and 30 μl LDH reaction mixture was added into each well. This was followed by incubation in a dark room at room temperature for 30 min. The absorbance at 490 nm was determined using a microplate reader (VersaMax Microplate Reader). According to the published equation (15), first, the difference in the OD_490_ values between experimental LDH release/spontaneous LDH release/maximum LDH release and blank control was obtained. Then the percentage of LDH release was calculated as follows: LDH release (%) = (experimental LDH release−spontaneous LDH release)/ (maximum LDH release−spontaneous LDH release).

***ROS assay***

The level of intracellular ROS generation was quantified using a Reactive Oxygen Species Assay kit (Beyotime, Shanghai, China). A fluorescent ROS-specific dye 2′,7′-dichlorodihydrofluorescein diacetate (DCFH-DA) probe was diluted with serum-free DMEM to a final concentration of 10 μM/l. After transfection with siPrdx1 and exposure to OGD/R, N2a cells were trypsinized with 0.25 % trypsin for 2 min and resuspended using diluted DCFH-DA fluorescent probe. Subsequently, N2a cells were incubated at room temperature for 30 min in the dark and then washed thrice with PBS to remove the extracellularly redundant DCFH-DA fluorescent probes. The resuspended N2a cells after centrifugation were evenly seeded in 96-well plates at 1 × 10^4^ cells per well. To obtain the intracellular ROS content, fluorescence intensity at an excitation wavelength of 488 nm and emission wavelength of 525 nm were measured using a multi-functional microplate reader (Bio-Tek, Winooski, VT, USA). The ROS production level was calculated in each group relative to the control group (16).

***Flow cytometry analysis***

Cell apoptosis was estimated using an annexin V/ propidium iodide (PI) cell apoptosis kit (Beyotime, Shanghai, China). After N2a cells were exposed to OGD/R for 8 hr, approximately 1 × 10^5^ cells per well were washed twice with cold PBS, trypsinized with EDTA-free trypsin followed by re-centrifugation and resuspended in 195 μl of Annexin V-fluorescein isothiocyanate conjugated binding buffer. Next, each brown EP tube was loaded with 5 μl of Annexin V and 10 μl of PI followed by incubation in the dark for 15 min. The proportion of apoptotic cells was detected by a flow cytometer (Mindray, Shenzhen, China) and analyzed using the FlowJo software. 

***Cell counting kit 8 (CCK-8) assay***

Cell viability after OGD/R exposure was estimated utilizing the cell counting kit-8 assay (CCK-8, Bioss, Beijing, China). Cells with a density of 4 × 10^3^ per well were seeded in 96-well plates (five replicated wells for each group). Wells with N2a cells that were cultivated under normal conditions were set up as the negative control and a well with medium lacking N2a cells was set up as blank control. OGD/R-exposed and unexposed N2a cells were treated with 10 μl/well of CCK-8 reagent in the dark for 2 hr. Cell viability was assessed at 450 nm (OD_450_) using a SpectraMax iD5 multi-mode microplate reader (Meigu Molecular Instrument Co, Ltd, Shanghai, China). According to the previous study (17), the cell viability was calculated as cell viability (%) = ([OD experiment − OD blank]/[OD negative control− OD blank]) × 100%. 

***Western blot assay***

Cellular protein was extracted by adding RIPA lysis buffer (Beyotime, Shanghai, China) containing protease and phosphatase inhibitors. The supernatant was aspirated after centrifugation (12,000 × g, 4 ℃, 20 min). The protein concentration was determined using the enhanced BCA protein assay kit (Beyotime, Shanghai, China). Equivalent protein concentrations per sample were separated using 10% SDS-PAGE and electrophoretically transferred to polyvinylidene fluoride (PVDF) membranes employing a semi-dry method. Thereafter, membranes loaded with phosphorylated or nonphosphorylated proteins were blocked for 1 hr with 5 % bovine serum albumin or 5 % non-fat milk, respectively at room temperature. Then, the membranes were incubated overnight at 4 ℃ with the following primary antibodies (monoclonal, rabbit, Abcam, Cambridge, UK): Prdx1 (ab211538, Lot# GR3416705, 1:1000), JNK (ab179461, Lot# GR244162, 1:1300), p-JNK (ab76572, Lot# 3431937-1, 1:1800), Cleaved caspase-3 (ab214430, Lot# 1018804-1, 1:1500), β-actin (monoclonal mouse, ab8226, Lot# 66009-1-Ig, 1:2000). Membranes were incubated with goat anti-rabbit or anti-mouse HRP conjugated antibodies (Abcam, Cambridge, UK) after rinsing for 1 hr at room temperature. Finally, the corresponding protein band was visualized using enhanced chemiluminescence (GE Healthcare Bioscience, Pittsburgh, USA), and the densitometry of the images was quantified and normalized to β-actin with Image-Pro Plus 6.0 software.

***Statistical analysis***

SPSS 22.0 software (SPSS Inc., Chicago, IL, USA) was utilized for statistical analysis. The results of the experimental data were presented in the form of mean ± standard deviation (SD). The comparison among the different groups was applied using the one-way analysis of variance followed by the* post hoc *test using the least significant difference. *P*<0.05 was considered statistically significant.

## Results

***Evaluation of transfection efficiency of Prdx1 siRNA in N2a cells***

**Figure 1:**
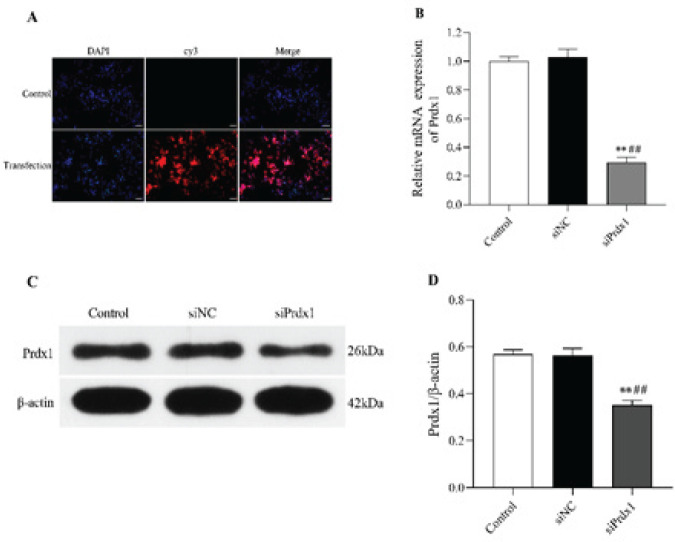
Evaluation of transfection efficiency of Prdx1 siRNA in N2a cells

Effective transfection of Prdx1 siRNA was essential to confirm the role of Prdx1 in OGD/R-exposed N2a cells. Herein, multiple detection methods were implemented to ensure the success of transfection. Fluorescence images were acquired under an inverted fluorescence microscope (Olympus Corporation, Hamburg, Germany) 48 hr after transfection. As shown in Figure 1A, Prdx1 siRNA was labeled with cy3 fluorophore (red), and nuclei were labeled with DAPI (blue). The transfected N2a cells emitted bright red fluorescence exhibiting a uniform distribution with the Cys-fluorophore-mediated fluorescence encircling the DAPI-mediated fluorescence. As a control, the normal N2a cells showed only DAPI-mediated fluorescence. The expression levels of Prdx1, including both mRNA (*P*<0.01, Figure1B) and protein (Figure 1C;* P*<0.01, Figure 1D) in the siRNA group dramatically declined when compared with those in the control group or siNC group at 48 hr following transfection. The outcomes demonstrated that the transfection of Prdx1 siRNA into N2a cells effectively down-regulated the expression of Prdx1.

***Prdx1 inhibition enhanced the level of JNK phosphorylation and caspase-3***

**Figure 2:**
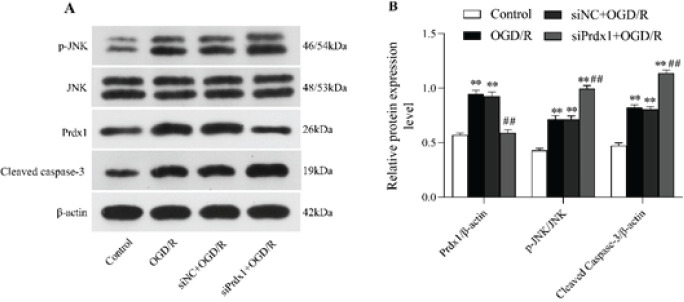
Prdx1 inhibition enhanced the level of JNK phosphorylation and caspase-3

To show the role of Prdx1 in OGD/R-induced injury, the activation of proapoptotic JNK/caspase-3 signaling cascades was evaluated by measuring the expression of p-JNK and cleaved caspase-3 using western blotting. As shown in Figures 2A and 2B, increased levels of Prdx1, p-JNK (relative to JNK), and cleaved caspase-3 expression were observed in N2a cells following OGD/R exposure (*P*<0.01). As expected, there was no difference between the OGD/R group and siNC+OGD/R group on those expression levels (*P*>0.05**)**. However, Prdx1 siRNA-mediated knockdown enhanced the expression levels of p-JNK/JNK and cleaved caspase-3 (*P*<0.01), whereas the expression levels of Prdx1 decreased when compared with those in the OGD/R group (*P*<0.01). These results suggested that knockdown of Prdx1 normally activated JNK phosphorylation and caspase-3 cleavage.

***Prdx1 knockdown aggravated N2a cells injury with OGD/R***

**Figure 3:**
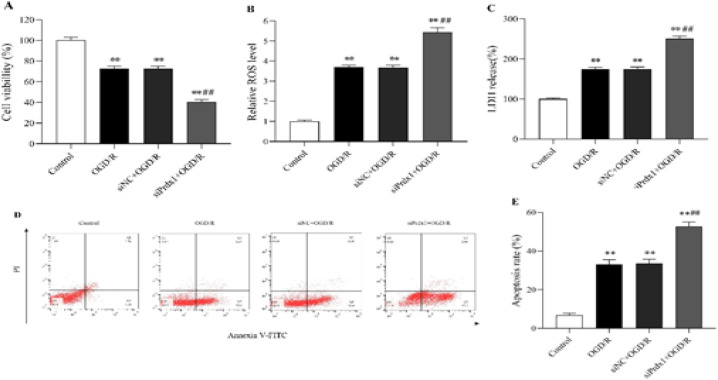
Prdx1 knockdown aggravated OGD/R-induced injury in N2a cells

Prdx1 loss-of-function experiments were conducted following Prdx1 siRNA transfection of N2a cells. The viability of OGD/R-exposed or unexposed N2a cells was measured utilizing CCK8 assay (18). LDH release assay was done to reflect the cytotoxicity (19). Cell viability decreased upon exposure to OGD/R, and Prdx1 siRNA further reduced the viability of N2a cells with OGD/R (*P*<0.01, Figure 3A). ROS and LDH release levels were also increased following OGD/R, and a further increase was observed after Prdx1 siRNA transfection (*P*<0.01, Figure 3B; *P*<0.01, Figure 3C). Likewise, when comparing the Control group, there was a significant increase in cell apoptosis rate in the other groups (Figure 3D; *P*<0.01, Figure 3E). The apoptosis rate was significantly promoted after transfection with Prdx1 siRNA, as tested by flow cytometry (Figure 3D; *P*<0.01, Figure 3E). Collectively, these data confirmed that knockdown of Prdx1 further aggravated the damage of N2a cells caused by OGD/R.

***JNK inhibitor SP600125 inhibited the JNK/caspase-3 signaling pathway and mitigated OGD/R-induced injury in N2a cells***

**Figure 4:**
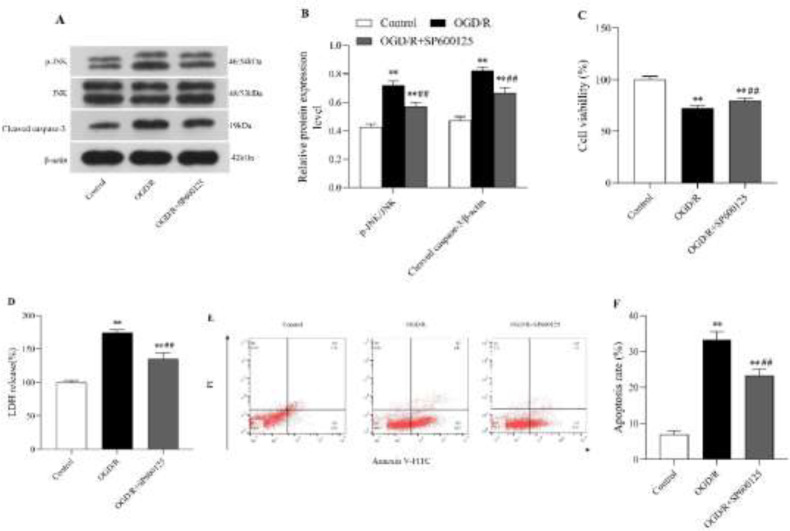
The JNK inhibitor SP600125 suppressed JNK/caspase-3 signaling pathway activation and mitigated OGD/R-induced injury in N2a cells

Figure 4A showed the effects of SP600125 (JNK-specific inhibitor) on the JNK/caspase-3 signaling pathway. Compared with the OGD/R group, the levels of p-JNK/JNK and cleaved caspase-3 proteins were significantly weakened by SP600125 in N2a cells following OGD/R (Figure 4A; *P*<0.01, Figure 4B). Meanwhile, SP600125 increased the cell viability (*P*<0.01, Figure 4C) and diminished both the LDH release (*P*<0.01, Figure 4D) and cell apoptosis (Figure 4E;* P*<0.01, Figure 4F) in OGD/R-induced N2a cells while compared with those in OGD/R group.

***SP600125 weakened the effects of Prdx1 siRNA on OGD/R-treated N2a cells***

**Figure 5:**
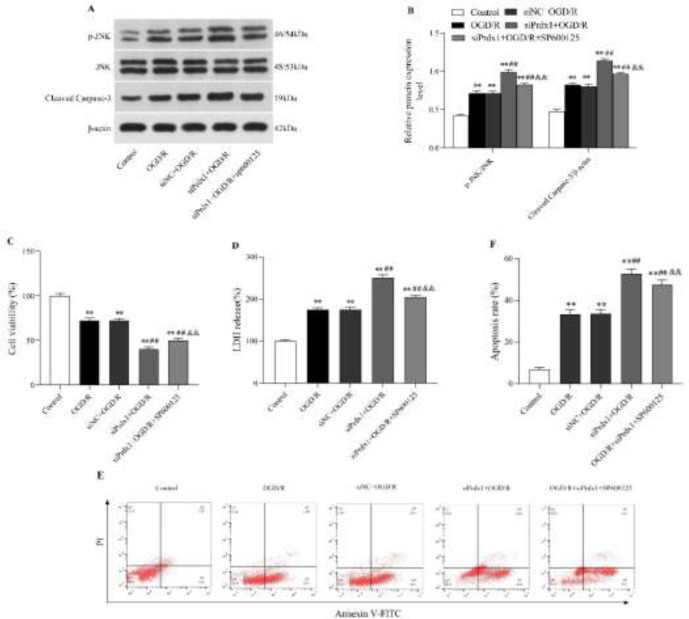
SP600125 weakened the effects of Prdx1 siRNA on OGD/R-treated N2a cells

The combined interventions of SP600125 and Prdx1 siRNA aimed to validate whether Prdx1 knockdown aggravated OGD/R-exposed N2a cells injury via activating the JNK/caspase-3 pathway. Prdx1 knockdown further increased the levels of p-JNK/JNK and cleaved caspase-3 compared with the OGD/R group or siNC+OGD/R group (Figure 5A;* P*<0.01, Figure 5B). Notably, after the combined use of SP600125, the activation of the JNK/caspase-3 pathway induced by Prdx1 siRNA following OGD/R exposure was largely eliminated (Figure 5A;* P*<0.01, Figure 5B). Moreover, the aggravated cells injury induced by Prdx1 siRNA was alleviated with the addition of SP600125 as shown by increased cell viability (*P*<0.01, Figure 5C), decreased LDH release (*P*<0.01, Figure 5D), and reduced cell apoptosis (Figure 5E; *P*<0.01, Figure 5F). Collectively, our results demonstrated that regulation of Prdx1 for OGD/R-induced N2a cell injury was implemented through JNK/caspase-3 signaling pathway.

**Table 1:** siRNA and primer sequences of Prdx1 and GAPDH

Name	siRNA or primer sequence
Prdx1 siRNA	5'-GCACCATTGCTCAGGATTA-3'
Prdx1	Forward Primer:5'-CTTCTGTCATCTGGCATGGATTAAC-3' Reverse Primer:5'-AAGACTCCATAATCCTGAGCAATGG-3'
GAPDH	Forward Primer: 5’-ACCCGCAGACCTCTCATTCT-3’ Reverse Primer: 5’-TGACAACGTTGGGTGAAAAA-3’

(A) The fluorescence images of N2a cells transfected with Prdx1 siRNA for 48 hr. The Control row (untransfected) shows the images of normal N2a cells, and the Transfection row shows the images of transfected N2a cells. The red fluorescence represents Prdx1 siRNA labeled with cy3 fluorophore. The blue fluorescence represents the nuclei labeled with DAPI. Scale bars = 50 μm. (B) qRT-PCR for Prdx1 mRNA expression. Relative quantities of Prdx1 mRNA normalized to GAPDH. (C) Western blot assay showing Prdx1 protein expression. (D) Relative quantities of Prdx1 protein normalized to β-actin expression (n = 5).

**P<0.01 versus Control group; ## *P*<0.01 versus siNC group

(A) Western blot assay for p-JNK, JNK, Prdx1, and cleaved caspase-3 protein expression in OGD/R-exposed N2a cells. (B) Relative expression levels of Prdx1, p-JNK, and cleaved caspase-3 proteins (normalized to β-actin expression; average of n= 5).

**P<0.01 versus Control group; ##* P*<0.01 versus OGD/R group

(A) Cell viability assessment using CCK-8 assay. The data in the control group was set at 100%. (B) Quantitative analysis of the ROS generation (fold change/control) using DCFH-DA. The data in the Control group was set at 1. (C) LDH release assay for LDH release. The data in the control group was set at 100%. (D) Flow cytometry analysis for cell apoptosis. The right quadrants (Q2+Q3) represent the apoptotic cells. (E) Quantification of the apoptosis rate (n = 5).

**P<0.01 versus Control group; ## *P*<0.01 versus OGD/R group

(A) Western blots showing p-JNK, JNK, and cleaved caspase-3 protein expression levels in control and OGD/R-exposed N2a cells. (B) Relative quantities of proteins (normalized to β-actin expression). (C) Cell viability measuring by CCK-8 assay. The Control group was set at 100%. (D) LDH release assay for estimating LDH release. The Control group was set at 100%. (E) Analysis of cell apoptosis by flow cytometry. Right quadrants (Q2 + Q3) represent the apoptotic cells. (F) Quantification of apoptosis rate (n = 5).

**P<0.01 versus Control group; ##*P*<0.01 versus OGD/R group

(A) Western blot assay for p-JNK, JNK, and cleaved caspase-3 protein expression in OGD/R-treated N2a cells. (B) Relative quantities of proteins normalized to β-actin expression. (C) Cell viability using CCK-8 assay. The data in the control group was set at 100%. (D) LDH release assay for LDH release. The data in the control group was set at 100%. (E) Flow cytometry analysis for cell apoptosis. Right quadrants (Q2 + Q3) represent the apoptotic cells. (F) Quantification of the apoptosis rate (n = 5).

**P<0.01 versus Control group; ##*P*<0.01 versus OGD/R group; && *P*<0.01 versus siPrdx1+OGD/R group

## Discussion

Timely vascular recanalization is an effective treatment against ischemic stroke (20, 21). However, blood flow recovery usually aggravates the existing brain damage, termed cerebral ischemic/reperfusion injury (CIRI) (22). Although multiple molecular mechanisms are known to contribute to CIRI, including excitotoxicity, oxidative stress, and inflammatory responses (23-25), the exact mechanism still needs further investigation. Mouse neuroblastoma cell lines (N2a) are widely used to study the pathophysiological mechanism of Alzheimer’s disease, nerve growth and regeneration, neurotoxicity due to its neural stem cell characteristics, ease of transfection, and abundant expression of tubulin (26). According to a previous study (14), N2a cells were exposed to oxygen–glucose deprivation/reoxygenation (OGD/R) to mimic the CIRI model in this study. The increased number of apoptotic N2a cells and increased expression of apoptotic components (cleaved caspase-3) indicated the successful establishment of these models.

Notably, the deleterious ROS production dramatically increased following reperfusion, which generated oxidative stress (OS) and disturbed the balance of oxidation and anti-oxidation, ultimately leading to cellular apoptosis, autophagy, or necrosis (6, 27). Excess ROS generation induced cell death via activating the proapoptotic signaling pathway, triggering DNA damage, lipid peroxidation, and loss of protein function during cerebral I/R (4, 28). Meanwhile, prior studies (29-31)  demonstrated that scavenging or regulating ROS would help to relieve cerebral I/R injury.

As a well-known member of the oxidative stress-inducible peroxidases family, peroxiredoxins (Prdxs) have been regarded as crucial anti-oxidant proteins responsible for developing a potent anti-oxidative defense system (32). Among the six subforms (Prdx 1-Prdx 6) (33), Prdx1 is necessary for cell survival, inflammatory response, and signal transduction in the central nervous system (CNS) under normal oxidative metabolism and oxidative stress (34-36). In our current study, the OGD/R-induced stress state of N2a cells triggered the expression of Prdx1 to reduce oxidation level which might be a self-protection mechanism. However, knockdown of Prdx1 interfered with this mechanism that further increased ROS levels following OGD/R.

The various functional effects of Prdx1 in the CNS have been extensively studied. In 6-hydroxydopamine (6-OHDA)-treated MN9D DA neuronal cells, it has been demonstrated that the generation of hydrogen peroxide and superoxide anion are largely blocked followed by decreased activated caspase-3 when Prdx1 is overexpressed. On the contrary, knockdown of Prdx1 could activate the 6-OHDA-induced apoptotic cell signaling pathway (37). Moreover, Prdx1 is also known as an important RNA-binding protein engaged in regulating inflammatory response and apoptosis in intracerebral hemorrhage rat models. (38). Recently, a study explored that maintaining the reduction activity of Prdx1-4 exerted a neuroprotective effect in rats with CIRI (39). Thereafter, it was reported that low expression levels of Prdx1 contributed to increased OS and cellular damage in OGD/R-exposed primary astrocytes (40). Similar neuroprotective effects of Prdx1 were also observed in both *in vitro* and *in vivo* models (8). In line with these findings, our study demonstrated that Prdx1 increased to combat OS injury. However, the targeted knockdown of Prdx1 further worsened the OS injury and caused a remarkable decrease in cell viability, increased LDH release, and cellular apoptosis. 

As a subfamily of mitogen-activated protein kinases (MAPKs), c-Jun N-terminal kinase (JNK) has three isoforms: JNK1(46kDa), JNK2(55kDa), and JNK3(48kDa). Extracellular stimuli phosphorylate JNK(p-JNK) and activate the JNK signaling pathway through mitogen-activated protein kinase kinase kinase (MAPKKK)-mediated phosphorylation and activation of MAPKK (41). JNK signaling pathway participates in the progression of multiple neuropathologies, including stress-induced cell inflammation, apoptosis, and necrosis during CIRI (42, 43). Previous studies have shown that, after CIRI or OGD/R injury, the increased p-JNK and cleaved caspase-3 were able to activate apoptotic-signaling cascades and induce ischemic cell death (44). Similarly, our outcomes showed an increased expression of p-JNK and cleaved caspase-3 in N2a cells exposed to OGD/R. However, SP600125 (inhibitor of JNK) decreased the level of p-JNK and cleaved caspase-3, and protected N2a cells against OGD/R injury, which was consistent with the earlier findings (45).

It has been reported that cytoplasmic Prdx1 has anti-apoptotic functions via direct or indirect interactions with JNK. As an endogenous regulator of JNK activity, Glutathione S-transferase pi (GSTP) was considered to bind JNK and suppress its activation.  Prdx1 could physically interact with GSTP and maintain the GSTP-JNK complex to inhibit the activation of JNK (46). Another possible and relevant pathway involves apoptosis signal-regulating kinase 1(ASK1), which belongs to a member of the MAPKs family and activates the JNK signaling cascade reaction. Prdx1 suppressed the activation of JNK by interacting with ASK1 on the thioredoxin-binding domain of ASK1 and inhibiting the phosphorylation/activation of ASK1 in human embryonic kidney 293 cells treated with H_2_O_2 _(47). A previous study demonstrated that Prdx1 could preserve the activity of embryonic stem cells by inhibiting the excessive generation and activation of ROS/JNK (48). Additional research showed that Prdx1 could suppress the activity of JNK, which resulted in decreased localization of forkhead box transcription factors of the O class (FOXO3) in the nucleus in H_2_O_2_-treated cells (49). ROS could dissociate the combination of GSTP and JNK (50), herein it could be assumed that the role of Prdx1 to scavenge ROS was probably in the stabilization of GSTP-JNK complex. In this study, JNK was activated although the expression of Prdx1 increased following OGD/R, which suggested that multiple and sophisticated factors may be involved in the regulation of the JNK signaling pathway. However, p-JNK was further raised after knockdown of Prdx1 which showed the inhibitory effects of Prdx1 on JNK activation. Interestingly, the combined use of JNK inhibitor partially rescued the outcomes of Prdx1 knockdown. This suggests that Prdx1 worked by suppressing JNK activation. 

This study had some limitations. The possible mechanism underlying the interaction of Prdx1 and JNK and the *in vivo* outcomes need to be identified and verified.

## Conclusion

The outcomes of the present study demonstrate that Prdx1 alleviates N2a cell injury with OGD/R via suppressing the JNK/caspase-3 pathway.

## Authors’ Contributions

GF Z and HP W designed the experiments; Y Y and HL C performed experiments; Y Y, JW Z, and HC T collected data; Y Y, GF Z, and HP W discussed the results and strategy; Y Y prepared the draft manuscript; F S, MS W, R D, GFZ, and HP W revised or edited the article; GF Z and R D supervised and acquired the funds. Y Y, HC T, HL C, JW Z, F S, MS W, GF Z, HP W, and R D approved the final version to be published.

## Data Availability

The raw data supporting the conclusions of this article are available from the corresponding author upon reasonable request.

## Conflicts of Interest

None of the authors have any conflicts of interest.
